# Understanding the Barrier and Mechanical Behavior of Different Nanofillers in Chitosan Films for Food Packaging

**DOI:** 10.3390/polym13050721

**Published:** 2021-02-26

**Authors:** João Pires, Camila Damásio de Paula, Victor Gomes Lauriano Souza, Ana Luísa Fernando, Isabel Coelhoso

**Affiliations:** 1 MEtRICs, Departamento de Ciências e Tecnologia da Biomassa, NOVA School of Science and Technology|FCT NOVA, Campus de Caparica, Universidade NOVA de Lisboa, 2829-516 Caparica, Portugal or victor.souza@inl.int (V.G.L.S.); ala@fct.unl.pt (A.L.F.); 2 Faculdade de Ciências Farmacêuticas, Universidade de São Paulo, Av. Prof. Lineu Prestes 580, CEP, São Paulo 05508-900, Brazil; camila.damasio@usp.br; 3 International Iberian Nanotechnology Laboratory (INL), Av. Mestre José Veiga s/n, 4715-330 Braga, Portugal; 4 LAQV-REQUIMTE, Departamento de Química, NOVA School of Science and Technology|FCT NOVA, Campus de Caparica, Universidade NOVA de Lisboa, 2829-516 Caparica, Portugal

**Keywords:** biopolymer, mechanical properties, bionanocomposites, food packaging, oxygen permeability, water vapor permeability

## Abstract

The continuous petroleum-based plastics manufacturing generates disposal issues, spreading the problem of plastic pollution and its rise in the environment. Recently, innovative techniques and scientific research promoted biopolymers as the primary alternative for traditional plastics, raising and expanding global bioplastic production. Due to its unmatched biological and functional attributes, chitosan (Ch) has been substantially explored and employed as a biopolymeric matrix. Nevertheless, the hydrophilicity and the weak mechanical properties associated with this biopolymer represent a significant intrinsic restriction to its implementation into some commercial applications, namely, in food packaging industries. Distinct methodologies have been utilized to upgrade the mechanical and barrier properties of Ch, such as using organic or inorganic nanofillers, crosslinkers, or blends with other polymers. This review intends to analyze the most recent works that combine the action of different nanoparticle types with Ch films to reinforce their mechanical and barrier properties.

## 1. Introduction

The food industry employs distinct types of materials in which glass and metal containers are highlighted for their excellent physical, gas, and water barrier properties, thus becoming important options to be considered by the food and beverage packaging. However, this type of materials have some negative aspects associated; they have increased transportation costs, demand excessive energy to recycle, and if not properly disposed of, may pose a risk to the environment because they do not decompose or decompose slowly in landfills or in the environment, releasing polluting substances [1]. Over the past century, plastic packaging has been increasingly replacing these traditional materials, due to their flexibility, variability in size and shape, thermal stability, and barrier properties. Despite all the appealing aspects undoubtedly connected to conventional plastic packaging, their non-renewable character, unsustainable use, and brief lifetime associated with their resistance to degradation caused a severe and realistic environmental problem, bringing out the responsibility to uncover creative and better ways to improve the disposal systems [2]. To fulfill the task of attenuating the ecological impact successfully and reduce the waste associated with the end of life of single-use plastics, novel bio-based food packaging systems should be intensely studied and optimized before being addressed for commercial purposes, ensuring that they are fully biodegradable and that no harmful toxicological and ecotoxicological effects arise from their use and disposal [3]. Bio-based plastics/polymers, recurrently expressed in literature as bioplastics or biopolymers, have recently been appointed as the logical candidates to replace conventional plastics due to their renewability, high abundance, accessibility, low cost, reduced toxicity, and biodegradable character [1]. The biopolymers normally used are polysaccharides (e.g., chitosan or pectin), lipids (e.g., natural waxes), proteins (e.g., whey protein), polyesters produced by microorganisms (e.g., polyhydroxybutyrate), synthesized from monomers (e.g., polylactic acid) or composites [4,5,6,7,8,9]. Chitin, a molecule derivative from glucose, is the second most plentiful natural polysaccharide found on our planet after cellulose and is mainly located in marine arthropods (i.e., more specifically in the exoskeleton of crustaceans) and in many other invertebrates, such as the cell walls of some fungi and algae. When submitting chitin to a fully or partially N-deacetylation, the acetyl group (–COCH_3_) from the amino group (–NH_2_) at the C-2 position is removed and a new bioactive polymer is designated as chitosan (Ch) arises. Structurally, Ch is a linear cationic polysaccharide with variable molecular weight (MW), assembled by D-glucosamine and N-acetyl-d-glucosamine units bonded by β-1,4 glycosidic linkages, with one amino (NH_2_) group and two hydroxyl (OH) groups in each repeating glycosidic units [10,11,12]. The ratio of D-glucosamine units to the sum of D-glucosamine and N-acetyl-d-glucosamine units that constitute the Ch chain is defined as the degree of deacetylation (DD). Pure Ch is insoluble in water, alkaline solutions, and even in organic solvents; however, when the DD reaches about 50%, it becomes soluble in slightly acidic environments (below its pKa ~6.3). As a result, Ch forms a highly versatile viscous solution after being dissolved due to the amino groups in the chain that protonate (–NH_3_^+^), increasing intermolecular electric repulsions and resulting in a polycationic soluble polysaccharide, allowing it to activate reactive locations for a variety of new group attachments using mild reaction conditions [11,12]. Films and coatings, specifically made of chitosan and derivatives, have been extensively studied in the past decades for fresh and processed food conservation [13,14,15] because they possess the beneficial aspects inherently associated with polysaccharides. The combination of these properties with the advantageous film-forming feature and antimicrobial/antioxidant activity unlocks the door to the introduction of suitable chitosan active films and coatings on valuable scientific and technological markets such as food applications, but also pharmaceutical and biomedical applications [16].

A correct evaluation of the polymer barrier and mechanical properties is crucial to estimate the product-package shelf life and its maneuverability. The permeability through plastic materials depends on several factors, namely, (i) the permeant properties (molecule size and nature); (ii) material/polymer characteristics (molecular orientation, crystallinity, free volume, and chain stiffness); and (iii) external conditions (such as temperature and moisture) [17]. The temperature and humidity circumstances to which the food product will be exposed in the supply chain are vital for calculating the required barrier so that it applies to the conditions expected. Furthermore, the specific barrier requirement of a package system depends upon the food characteristics and the intended end-use applications [17,18,19,20]. The two main permeants studied in food packaging applications are water vapor and oxygen, because those gases can move from the internal or external environment through the polymer package wall, they result in possible negative changes in product quality and shelf-life and in the polymer itself. Moreover, the study of the permeability of oxygen (OP) is important because the oxidation is related to the odor, color, taste, and deterioration of food; therefore, packages must provide a barrier to this gas [18,21].

Petroleum-derived plastics often present a good water vapor barrier but poor oxygen block properties. On the contrary, the biopolymeric matrix (i.e., chitosan) is generally attractive due to its good obstacle characteristics against oxygen. In this subject, due to the well-ordered hydrogen-bonded network configuration, polysaccharides unveiled to be an effective barrier to gas transference, such as O_2_ and CO_2_, preventing oxidative rancidity, and surface-browning. In contrast, they typically own a hydrophilic nature resulting in poor water vapor and moisture barrier properties, which can turn into a dilemma in the preservation of perishable fresh-cut products [11,19,20,21]. Together with the barrier properties, mechanical properties (characterized by tensile strength, elastic modulus, elongation at break) also play an important role in the development of novel biodegradable plastic materials. When compared to plastics, bioplastics still present critical structural flaws coupled with processing challenges, which diminish their industrial applicability [22]. Blends of chitosan and anionic polymers could be a solution, showing improved mechanical and barrier properties when compared to chitosan films. This can be attributed to the formation of polyelectrolyte complexes through electrostatic interactions between the protonated amino groups of chitosan and the negatively charged side-chain assemblies of the other biopolymer. Better performance in terms of water vapor permeability and lower water solubility have been reported for combinations of chitosan with other polysaccharides, such as starch, pectin, or alginate compared to chitosan films [20].

To make it possible for biopolymer-based food packaging to go from concept to reality, it is fundamental to strengthen their composition, modifying and improving their properties. Therefore, another undergoing solution is the use of organic or inorganic nanoparticles to enhance the properties of the films, as was demonstrated in the study by Jamróz et al. [23]. The insertion of homogeneously dispersed nanoparticles, with a high aspect ratio and high surface area, into the biopolymer matrix is seen as a promising innovation, creating a novel functional class of materials, named bionanocomposites [2,11,24,25]. Polymer nanocomposites are mixtures of a certain polymer (or biopolymer) reinforced with small quantities of nanosized inorganic or organic (or bio-based organic) nanofillers with a particular size, geometry, and surface chemistry [26]. Nanotechnology represents the manipulation of matter with at least one dimension sized from 1 to 100 nanometers, and nanomaterials are characterized for having different physical, chemical, and biological properties than bulk material [18]. Thus, nanotechnology plays an important role as an alternative strategy to reinforce bio-based films through the incorporation of nanomaterials into the polymeric chain to enhance their mechanical and barrier properties [27]. After being reinforced with nanofillers, the chitosan-based nanocomposites are capable to be applied in various scientific areas and industries such as paper and food packaging, flexible electronics, sensing and biosensing, energy harvesting, liquid crystals, biomedicine and cosmetics, catalysis, adsorption, separation, decontamination, or filtration systems [28,29,30,31,32,33].

This review’s main focus is to explore and summarize the latest results on how different nanofillers interact with chitosan in order to enhance the film’s barrier and mechanical properties to be used efficiently in food packaging systems. Critical analysis of the most recent information available will be useful to identify future opportunities for the use of nanoparticles in food packaging systems, helping to overcome current restrictions associated with the limited scale-up of chitosan-based polymers in food packaging.

## 2. Nanotechnology as Reinforcement

### 2.1. Barrier Properties

The incorporation of nano-sized particles is under intense investigation for enhancement of barrier properties of packaging. The barrier properties of polymers can be significantly altered by the inclusion of inorganic platelets with a high aspect ratio to alter the diffusion path of gas molecules. Various models have been proposed to predict the permeability of platelet-filled composites as listed in Table 1. These models are generally based on random, parallel platelets perpendicular to the permeation direction (random in only two directions). At a high aspect ratio, significant decreases in permeability are predicted and observed in practice [34].

Common fillers used to prepare nanocomposites for food packaging applications are nanoclays, cellulose nanofibers and nanocrystals, carbon nanotubes, and metal oxides. Table 2, Table 3 and Table 4 show the effect of different nanofillers and compositions on water vapor permeability (WVP) and oxygen permeability (OP) through chitosan nanocomposite films.

The amount of nanofillers ranges between 0.5% (*w*/*w)* and 10% *w*/*w* of chitosan, except in a few cases in which concentrations are up to 30% (*w*/*w)*. It can also be seen that most studies present only WVP results. Although OP is an important parameter because it will influence the oxidation of the food products and consequently, the product shelf life is seldom reported [35].

In most studies, a decrease of permeability with the increase of filler amount may be observed. This is due to the increase in the diffusion path that slows the permeation of gases through the polymer barrier. In some, this trend happens until a critical nanoparticle content, after which a constant or a small increase of permeability is detected. This behavior has been explained with deficient nanoparticles’ exfoliation or a decrease in aspect ratio. Moreover, when inorganic particles are included in a polymer matrix, their effect on the composite permeability is related to the type of interaction of the particles with the permeant species, and with the polymer and the resulting internal morphology. For instance, regarding the barrier to water vapor, the water adsorbs to the polymer and hydrophilic nanoparticles. The swelling of both solids may create unfilled zones around the particles where water vapor diffusion will be made easier and higher. From a given particle content, the increase in the diffusion path due to the impermeable barrier is nullified by the contribution of these unfilled diffusion zones, leading to stability or even an increase in permeability [17].

The barrier properties of chitosan films with different nanofillers are presented and discussed in the following subchapters.

#### 2.1.1. Nanoclays

Montmorillonite (MMT) is a hydrated, alumina–silicate layered clay, and its negative charge is balanced with exchangeable cations, as Na^+^ and Ca^2+^. It is one of the most used clays because of its low cost, high active surface, and its capacity to improve the mechanical and barrier properties of chitosan nanocomposites [11]. The use of nanoclays in films has several advantages due to its low cost, availability, and good surface area. The addition of montmorillonite gives the films a lighter composition and less risk of microbial contamination, for the packaging and for the food packaged by this film, improving the shelf life of the food packaged [36]. Regarding toxicity, the MMT does not appear to be toxic and can be used widely in the food industry [37].

Nanoclays can provide the enhancement of the water barrier properties of bio-based films by the increase on the tortuous path of the water vapor molecules through the polymeric chain [38]. Exfoliated structures, in comparison with the intercalated and micro-phase separated structures, are more efficient to prevent the film from being water-permeable [11,38]. Because the permeability to water vapor is related to the deterioration of food, films intended to be in direct contact with food must have a good barrier to this water vapor [21]. Some examples of reinforced chitosan films with MMT or halloysite nanotubes (HNT) are shown in Table 2 and described below.

Films prepared with 1% (*w*/*v* Ch), 30% glycerol, and two different MMT at 0.5 (*w*/*w)* and 1% (*w*/*w* Ch), demonstrated a decrease in WVP proportionally to the amount of MMT added in the matrix (Table 2) [19]. According to Llanos et al. (2018) [42], this phenomenon occurred due to the tortuous path provided by the MMT nanoparticles that are dispersed in the chitosan matrix, which makes the water vapor more difficult to diffuse through the film. The WPV was determined using the gravimetric method. This study also evaluated the material’s oxygen permeability and the presence of nanoclay. Contrary to what was expected, the OP increased, because the nanoparticles (NPs) increased the porosity of the film, changing its microstructure [42]. The addition of MMT can improve the barrier properties of films; however, this nanoclay generally does not enhance the antimicrobial properties of chitosan films. Thus, it can be interesting to add antimicrobial agents, such as metal oxide nanoparticles or essential oils, to confer this antimicrobial activity to the packaging [11]. Copper oxide nanoparticles are known for their antimicrobial activity; however, the release of copper ions toward the food can cause leaching, enhance the food deterioration process, and pose some toxicity. Thus, the combination with MMT with exchangeable positively charged ions, such as Na^+^ or Ca^2+^, can prevent copper ions from being released [41].

Bionanocomposites based on chitosan (2% *w*/*v*) were formulated with the addition of 25% (*w*/*w* Ch) of glycerol and three different MMT concentrations (1%, 3%, and 5% *w*/*w* Ch) with or without CuO NPs. Two complexes were studied MMTCuO-20 and MMTCuO-90 (produced differently regarding the synthesizing time inside the muffle furnace, 20 min or 90 min, respectively) [41]. In this case, the WPV study was also conducted by the gravimetric method and the OP study by the ASTM D3985-05 [46] method. Overall, the addition of pure nanoclay decreased the WVP in all treatments; however, the lowest result was achieved with the addition of 3% of MMT (a decrease of 37% in comparison to the control sample). At the level of 5% MMT, the WPV was slightly superior to the composites with 3%, which was related to the possible agglomeration of the NPs. When the complex with MMT and CuO NPs were used, the same pattern was observed; however, CuO NPs were able to reduce even more the material WPV (reaching 77% reduction), and MMTCuO-90 at 3% was the best formulation. These results were attributed to the presence of hydrogen interactions between chitosan, glycerol, and an MMT–CuO, which decreased the number of hydroxyl groups in the film to react with the water, and the building of a tortuous path, that makes the diffusion of water vapor difficult [41]. Concerning OP, the addition of MMT to the matrix formed by chitosan and glycerol also reduced the oxygen permeability. The best result was also with the addition of MMT–CuO because the groups present in chitosan have a strong attraction with MMT–CuO, which closes the net of the film, preventing the entry of oxygen [41].

Contradictory behavior is also found in literature, as in Souza et al. (2019) [25] and Xu et al. (2018) [37], which did not observe a statistical difference in the WVP of chitosan films incorporated with different amounts of MMT (varying from 0.5–2.5% *w*/*w* Ch). However, the OP in both studies was reduced around 32–61% in comparison to pristine chitosan film. As described before, it seems that when the amount of MMT incorporated is increased the enhancement ability diminishes, probably due to the agglomeration effect. In addition, within the formulations assessed, the incorporation of 1% of MMT was demonstrated to be the best reinforcement formula [25,37].

Halloysite nanotubes (HNTs), Al_2_Si_2_O_5_(OH)_4_·nH_2_O, belongs to a subgroup of kaolin clay, which is generally recognized as a safe (GRAS) food packaging material due to its non-toxicity and biocompatibility [40]. HNTs show a nanotubular morphology, with an outer tube diameter of nanometric dimension, whereas the length can range typically from hundreds of nanometers to about 1.5 µm [40]. Some of the advantages of HNTs are their hydrophilic characteristic, high dispersibility (easily exfoliated because of its structure with high aspect ratio), thermal stability, and non-toxicity [40,47].

In vitro and in vivo studies with HNTs demonstrated their biocompatibility with tissues and cells, thus showing that they are also interesting options for film production. In the formulation was added 0.2% of chitosan, carboxymethyl cellulose (CMX) in the proportion of 1:2 to chitosan and HNT in three different concentrations, 5%, 7.5%, and 10% (*w*/*w* Ch) [44]. The authors concluded that with the addition of HNT, the chitosan and CMX films have a greater barrier to water vapor due to the dispersion of the nanoclay in the mixture. This phenomenon was attributed to the more tortuous paths due to the hydrophilic interaction between chitosan and HNT, which also caused the reduction of the hydrophilic parts, thus making the interaction with water vapor more difficult and, consequently, the diffusion process more difficult to occur [44].

#### 2.1.2. Cellulosic Nanofibers and Nanocrystals

Nanocellulose (NC), either as nanofibers or nanocrystals, are also nanomaterials commonly used to strengthen bio-based films by the improvement on their mechanical properties and the reduction on the WVP and OP [48], which are the main drawbacks of biopolymers’ application in the packaging industry [27]. They are an environmentally friendly nanofiller that can be highlighted due to their biodegradability, low density, nontoxicity, excellent mechanical properties, and high aspect ratio that guarantee a huge interface with the polymeric matrix [49,50,51].

As previously described for the nanoclays, the decrease in the WVP or OP is generally attributed to the tortuosity created by, among other factors, the incorporation of nanocellulose, and this reinforcement is a topic that merits further research attention [52]. The types of nanocellulose used for this purpose vary from the source, namely, bacterial or extracted from biomass, and configuration (in crystals or fibers). The content of NC used in the film is generally low, between 0–35% (*w*/*w* of polymer), as described in Table 3.

The trend observed is that despite the type or source of NC used, WVP reduces with the incorporation of nanocellulose particles until a maximum (in general below 10% *w*/*w*), from which with the increase of the amount incorporated the barrier properties reduces. In the study of Corsello et al. (2017) [53], bionanocomposites based on chitosan incorporated with cellulose nanocrystals (CNC) at four increasing levels (1%, 3%, 5%, and 10% *w*/*w* Ch) showed a reduction in the WVP up to 38% in comparison to pristine chitosan films. The minimum permeability to water vapor was observed with the amount of 5% CNC, and when 10% was incorporated, the WVP decreased to 25%. The author attributed the WVP reduction to the presence of NC, which increased the tortuosity in the polymer matrix films, leading to slower water vapor diffusion processes [53,55]. In contrast, excessive NC destroyed the polymeric network structure of chitosan, allowing for the formation of gaps for water vapor molecules to pass through [56].

Other factors might account for this reduction in the WVP, such as (i) the increase in the polymer’s crystallinity (acting as crystalline fillers that increases the distance for the molecules of water to pass through, i.e., a more tortuous path); (ii) the improvement on the film uniformity, and iii) making the film tough enough to decrease the frequency of cracks or other defects that could lead to a high gas permeability [52,56].

The study of OP is scarce, and few studies investigate it simultaneously with WVP, as in the study of Zhang et al. (2021) [56]. The author proposed a novel bionanocomposite of chitosan reinforced with curcumin grafted TEMPO-oxidized cellulose nanofibers (CGTOCNF) at 10%, 17%, 25%, and 33% (*w*/*w* Ch). Regarding WVP, similar behavior was reported, with a reduction in WVP with CGTOCNF incorporation. On the other hand, OP of the bionanocomposites increased with the incorporation of the nanoparticle complex; thus, the addition of CGTOCNF had a more powerful destructive effect than improved crystallinity for the polymer network, and the hydrophobicity of CGTOCNF was not sufficient to counterbalance this, contributing less to the oxygen barrier [56].

#### 2.1.3. Metal Oxides Nanoparticles and Carbon Nanotubes

Metal oxide nanoparticles and carbon nanotubes are other classes of NPs that can be used to improve the quality of the films in relation to their oxygen and water vapor barrier properties. Some examples of the latest studies on this matter are shown in Table 4.

According to the study by Sanuja et al. (2015) [59], who evaluated the WVP of chitosan films incorporated with three different concentrations of zinc oxide nanoparticles (ZnONP) (0.1%; 0.3%, and 0.5%), there is a decrease in the material’s WVP with the increment of the NP concentration in the formulation (up to 56% lower than pristine chitosan films). This behavior was attributed to the fact that chitosan has high hydrophilic properties, so it is easier to interact with the hydrogen in the water molecule, and with the addition of ZnO NPs, its dispersion in the matrix forms a more efficient barrier to contain the water vapor permeability. In this experiment, neem essential oil was also added to the chitosan formulation with 0.5% ZnO NPs. Because it presents even more hydrophobic characteristics and interacts with chitosan, it reduced WVP even further [59].

The use of ZnO NPs is very efficient to increase the antimicrobial properties of the film and its barrier properties, including WVP and OP, and that is the reason many studies with the reinforcement of chitosan with this NP are found in the literature. Moreover, ZnO NPs have antioxidant properties and are non-toxic when added to the film, being recognized as GRAS by the US Department of Food and Drug Administration (FDA). Chitosan films with ZnO NPs and gallic acid allowed the reduction of WVP in relation to the pure chitosan film, according to the study published by Yadav et al. (2021) [60]. This occurred due to the construction of complex paths for the passage of water and the occupation of spaces (porous) in the macromolecular structure. Concerning oxygen permeability, with the addition of ZnO NPs, the OP values reduced up to 41% when compared to pristine chitosan film [60].

In another study also using ZnO NPs to improve the barrier properties of the chitosan film, a decrease in WVP with the addition of ZnO NPs was reported [64]. According to the authors, when ZnO NPs are incorporated, structures with greater firmness are formed due to the interaction of the metal oxide NP and chitosan, generating a more complex path for the water molecules to diffuse through. In this experiment, silver nanoparticles (AgNPs) were also added to the formulation, i.e. composites of Chitosan-AgNPs and Chitosan-AgNPs-ZnNPs were evaluated. The addition of both nanoparticles to chitosan also showed satisfactory and better values than just the use of one of the compounds. Moreover, they also included citronella essential oil, and this formulation containing the four components presented the lowest value of WVP [64].

The use of carbon nanotubes (CNT) in the formulation of bio-based films was reported by Liu et al. (2019) [58]. The authors combined CNT with a polymeric blend of chitosan (in different percentages 1–9% *w*/*v* film-forming dispersion) and polylactic acid (PLA). When CNT was added to the formulation with chitosan, the resistance of the formed film was 90% greater than the pristine chitosan film, which corroborates with the WPV results. The lowest value of WVP was acquired when the amount of chitosan was 7% (*w*/*v* film-forming dispersion). This decrease in WVP was possible due to the reduction in the pore diameter of the fiber, making it difficult for water molecules to pass. In addition, because of the hydrophilicity, there was a certain absorption of the water vapor by the chitosan. However, if the amount of chitosan increases a lot, this hydrophilic characteristic can damage the water vapor barrier, and this may be one of the reasons related to the increase in WVP by adding 9% (*w*/*v* film-forming dispersion) chitosan in the formulation. In addition, the viscosity of the film can increase much more with the greater addition of chitosan, making it very porous and impairing its quality [58].

In another study that addresses chitosan films together with carbon-based coatings, no significant difference was observed in relation to the WVP. However, concerning OP, there was a decrease in this permeability when the addition of carbon-based coatings occurred, according to Fernandes et al. (2018) [62]. For this research, three different acetylene fluxes in standard cubic centimeters per minute (4 sccm, 12 sccm, and 20 sccm) were used to apply the CNT coating. The lowest OP value was acquired with the largest acetylene flux, 20 sccm, due to the presence of less porosity in the film. This is because the carbon coating reduces the affinity between the oxygen and chitosan molecules. The difference between the permeability of the film for water vapor and oxygen can be explained due to the high solubility of chitosan in water, which promotes diffusion with the micro-cracks that may exist in the film, and because the O₂ molecules do not degrade chitosan, this allows it to have greater coverage of the surface by the carbon coatings, having a greater barrier to oxygen [62].

### 2.2. Mechanical Properties

#### 2.2.1. Nanoclays

The application of layered clay minerals with large specific surface area, such as montmorillonite (MMT), as nano reinforcement in biopolymeric matrices, has been extensively explored, giving rise to numerous studies that demonstrate its ease to blend and consequent mechanical behavior improvement [66,67,68,69].

The manner that polymer and nanoclay are held together directly rules the nanocomposite physical and mechanical properties. To be considered effective it is recommendable to achieve a nanocomposite where the two components (chitosan and MMT) are perfectly intercalated or exfoliated, therefore avoiding a tactoid blend. When the polymeric chains accurately diffuse through the nanoclay layers, disrupting the van der Waals forces that hold the MMT stacks together and consequently increasing the interlayer spacing and forming a well-ordered multilayer structure, an interleaved composite can be accomplished. The polar interactions between the negatively charged silicate layers and the chitosan polycationic essence in acidic media predict a favorable interconnection through the cationic exchange process [70]. More specifically, the surface negative charge instability generated by isomorphous substitution that occurred in the octahedral sheets is counterbalanced by some commutable cations (majorly Na^+^ and Ca^2+^). In the presence of those cations adsorbed on the silicate layer, the cations inside the gallery can be readily exchanged by other cations [71,72]. Another type of nanocomposite, known as an exfoliated blend, is achieved if the clay sheets are completely separated and homogeneously dispersed in the matrix, increasing the polymer/clay interactions. If none of these composites types takes place, it leads to a non-desirable tactoid mix, where the MMT layers did not separate. In this case, complete particles will be dispersed within the polymeric matrix, diminishing the composite mechanical potential [71].

The assessment of specific mechanical parameters is essential to determine the viability of the polymer-based composite materials. By mixing the polymer matrix with the nanoparticles is projected the achievement of a nanocomposite with refined mechanical properties, structure, and integrity. The parameters that better express that evaluation are the maximum tensile strength at break (TS), the elongation at break (%EAB), and the elastic or Young’s modulus (EM). TS specifies the film strength, %EAB indicates the material’s deformation capacity, and EM measures the film rigidity [16]. Regarding chitosan films reinforced with MMT, it is properly settled by the literature that the nanoclay amount should not exceed 5% (*w*/*w* Ch), as expressed in Table 5. Up to this concentration is possible to observe an increment in terms of composite strength and rigidity, and in the counterpart, a decrease in the flexibility is perceived, a consequence of limited mobility of the polymer chains in the presence of clay. With the nanofiller load overcoming the 5%, the obtained materials become brittle, noting a significant decline in mechanical properties [73]. The constatation of the drop in TS for higher concentrations of MMT may be caused by some aggregation of nanoparticles with high surface energy [66].

The effectiveness of MMT’s performance as a mechanical reinforcement in a chitosan biofilm does not depend only on the amount added or on the affinity between them. In 2009, Hong et al. [71] stated that higher values of tensile strength were reached for 3% and 5% (*w*/*w* Ch) MMTNa^+^ contents, and in contrast, lower values for elongation at break as expected. At lower concentrations, the nanoclay dispersed more uniformly through the chitosan matrix, increasing the surface attraction between the two components. In addition to the effect of the nanoclay concentration on mechanical properties, the same study also tried to understand the effect of the mixture shear rate. The authors observed that the tensile strength of the films increased until a shear rate of 16,000 rpm and it did not change significantly for superior values [71]. Later in 2011, Potarniche et al. [77] tested different pH values and temperatures to study the effect of these two parameters on the MMT dispersion. It was verified that the silicate layers are better intercalated when blended with chitosan at higher temperatures and pH 5. It was supposed that with these conditions the chitosan chains are oriented preferentially between the silicate layers. Giannakas et al. (2014) [74] compared the mechanical properties of films reinforced with MMTNa+ with two different chitosan dilutions in acetic acid (1% and 2% (*w*/*v*)), and the compatibility with glycerol, used as a plasticizer. Through this study, it was possible to obtain pivotal conclusions about the interaction between the different parts that formulate the nanocomposite. When diluted from 2% to 1% (*w*/*v*), the film stiffness and strength were reduced, and an increase in the elongation at break was observed. The results could be explained by higher crystallinity and less charged amine groups present in more diluted solutions. Additionally, it was constated that the addition of glycerol helps in the opening of the nanoclay structures, and with that, the composites will become more plastic, with reduced stiffness and strength and higher elongations. Souza et al. (2018) [75] incorporated different commercial MMT’s in chitosan films at the level of 2.5% (*w*/*w* Ch). All nanoclays improved the mechanical properties with the two natural ones, MMTNa^+^ and MMTCa^++^, showing the most promising results [75].

To employ this modern range of materials as an active part of the packaging for the food industry, the introduction of essential oils has been tested to increase the film’s antimicrobial and antioxidant capacity [11]. These novel active compounds present in nanocomposites will in turn interact with the polymer and MMT. Abdollahi et al. (2012) [72] bound rosemary essential oil (REO) at three different levels with chitosan/MMT composites and the interaction between them was investigated. When compared with the same films without REO, it was retained that the interaction between them improved both strength and elongation at break, especially for REO at 0.5% (*v*/*v* film-forming dispersion). In contrast, Souza et al. (2019) [25] reported that chitosan/MMT films with ginger essential oil (GEO) were less resistant and rigid, and more plastic. The authors justified this with the formation of heterogeneous and discontinuous structures promoted by the incorporation of lipids into the biopolymeric matrix. Therefore, the strong polar chemical bonds chitosan–chitosan will be replaced by weaker interactions between chitosan–GEO. Giannakas et al. (2020) [76] observed similar results when introduced thyme oil in two different chitosan/MMT structures. As expected, they noted that the introduction of nanoclays, MMTNa^+^ and OrgMMT, improved mechanical behavior. However, these good results were lowered when the thyme oil (TO) was added, acting as plasticizer when they are directly added to the chitosan matrix. Instead of adding directly the TO into the film, the authors tried another method where the essential oil was firstly adsorbed into the MMTNa^+^ or OrgMMT, resulting in new Ch solution hybrids that exhibited higher performances. In this study, the authors suggested that the methodology used to incorporate the essential oil in the composite is fundamental for the interaction with chitosan/MMT, ruling the film’s physical properties [76]. Ghelejlu et al. (2016) [39] perceived similar results when characterized chitosan/MMTNa^+^ bionanocomposites loaded with milk thistle extract. Moreover, it should be noted that they only observed a significant decrease in mechanical properties for extract concentrations superior to 1% [39].

Crosslinking is another essential method to improve polymer mechanical properties. To date, few articles have explored the synergistic effect resulting from the affinity between MMT and the crosslinker, because it is difficult to understand the influence of the two components together on the mechanical properties of the films. Despite not having performed mechanical tests, Gierszewska et al. (2019) aimed to examine the influence of MMT and glutaraldehyde (GA) as crosslinker into the chitosan matrix [78]. The results suggested that the introduction of GA had a positive effect on MMT dispersion and, with that, a nanocomposite with superior physicochemical properties was obtained. However, it is impossible to determine with certainty in which way this affects mechanical behavior. Liang et al. (2019) [79] developed a chitosan nacre-like nanocomposite reinforced with MMTNa^+^ and genipin (GP), a naturally occurring crosslinking agent. The films that contain GP exhibited higher values of strength and stiffness compared with the films that only contain MMTNa^+^, with the tensile strength remarkably increasing by 60% and 255% when compared to pristine chitosan. This could be mostly attributed to the crosslinked networks formed by the Schiff reaction between GP with chitosan and not to a direct interaction between GP and the nanoclay, even because the crosslinking effect reduce the free movement of the polymer molecular chain, thus diminishing the degree of hydrogen bonding between CS and the MMT sheets [79].

It can be concluded that the addition of MMT nanoparticles has a beneficial effect on the mechanical properties of chitosan films. This improvement is mostly seen for concentrations between 3% and 5% (*w*/*w* Ch). However, other external factors such as the addition of plasticizers, crosslinkers, extracts, and essential oils can modify, increasing, or decreasing the nanoclay performance.

#### 2.2.2. Cellulosic Nanofibers and Nanocrystals

Cellulose in the form of nanofibers (CNF), nanocrystals (CNC), bacterial nanocellulose (BNC), or simply nanocellulose (NC) is the most available natural biopolymer on the planet Earth, but only lately has earned the reputation as a biodegradable nanoscaled material. These natural nanoparticles are equipped with unique optical, rheological, chemical (abundant hydroxyl groups) and, mechanical properties (high strength and high specific surface area) at low concentration, emphasizing them as a suitable agent for strengthening chitosan films [80]. Moreover, when compared to the established nanofillers, such as nanoclays, aluminum oxide particles, silica, mica, and carbon nanotubes, NC exhibits fewer public health risks [81]. The effects resulting from the incorporation of nanocellulose in composites are found in a considerable number of quality studies. The majority of them take into account NC directly hydrolyzed from pure sources, such as chemically treated microcrystalline cellulose or cotton [50,54,82,83]; yet, a recent interest is being demonstrated about the NC properties produced from lignocellulosic residues [84,85,86,87].

From the studies analyzed in this review, a consensus among researchers is evident that 5% of nanocellulose is enough to be considered the most suitable percentage to mechanically reinforce Ch films. Still, in 2010, Azeredo et al. [55], when evaluating the synergy between CNF and glycerol added to chitosan films to improve its mechanical and barrier properties, verified that the optimum conditions were the addition of precisely 15% CNF and 18% glycerol. Later, Gopi et al. (2019) [88] studied the incorporation of cellulose nanofibers extracted from turmeric residue into Ch films at four distinct levels (1–7% (*w*/*w* Ch)). The tensile strength and Young’s modulus registered an increase for all concentrations, reaching the highest rise of 31% and 18%, respectively, for a 5% CNF amount. The interlinkage of CNF by hydrogen bonding with Ch generated an ably reinforcing fibrillar network, which upgraded the mechanical properties considerably. In the same year, Jacob et al. [89] and Kumar et al. [90] used similar CNF levels as reinforcement, but in this specific matter, the cellulosic nanofibers were extracted from ginger rhizomes and jute fiber, respectively. The results adopted the trend of Gopi’s research, but with more significant improvement percentages. Of note, the superior strength and stiffness values obtained by Jacob et al. (2019) [89], around 126% and 89%, reached for the 5% CNF. In this particular, CNF level, it was stated predominant and most intense intermolecular hydrogen bonds between the bionanocomposite components, data supported by the FTIR results exposed in the same study. Kumar et al. (2019) [90] also complemented, expressing the minor size and uniform distribution of CNF in the chitosan matrix could mean a minimization of pours and failure spreading points. Furthermore, for both studies, the %EAB diminished with the nanocellulose incorporation, as expected.

When compared to the Ch/CNF, the Ch/CNC bionanocomposites seem to indicate similar mechanical behavior. Khan et al. (2012) [91] prepared through solution casting chitosan-based biodegradable films reinforced with four different concentrations of nanocrystalline cellulose (1–10% (*w*/*w* Ch)). The most noticeable mechanical improvement was attributed to films with 5% CNC, in which the tensile strength improved 25% and the tensile modulus 87% in comparison to pristine chitosan. The explanation behind the favorable chitosan–nanocellulose interactions could reside in the good interface between the anionic sulfate groups of CNC and the cationic amine groups of Ch. Beyond this CNC amount, a potential aggregation of nanoparticles occurred that did not help to improve the mechanical properties. In opposed tendency, was verified a slight decrease relatively to elongation at break. This decrease suggested a tight interaction between filler and matrix, which narrowed the matrix mobility, decreasing the %EAB [91]. To value agricultural waste, Xu et al. (2018) [86] isolated cellulose nanocrystals from rice straws and used them to develop high-value bionanocomposites. Following the previous research route, the authors observed a constant increment in the tensile strength until it reaches the optimal CNC value of 5%. In addition to the potential occurrence of nanoparticle aggregation that could explain the weak interfacial Ch/CNC compatibility after reaching the optimal amount of reinforcement, this study also attributes to the charge neutralization with poor dispersion between –COO^−^ and –NH_3_^+^ moieties. On the other hand, the %EAB proportionally decreased with the rise of the CNC content [86]. Other studies, shown in Table 6, aid to conclude that the ideal CNC incorporation level to grasp the upper-interfacial compatibility should be around 5%, curiously the same amount perceived for MMT. This percentage allows achieving optimal nanocellulose dispersion, robust hydrogen bonding, and feasible electrostatic interactions between Ch and CNC. Interestingly, the work of Mujtaba et al. (2017) [84] has slightly deviated from the formerly exposed tendency. This research was focused on the CNC extraction from flax fibers, which were later used in higher percentages (5–30% (*w*/*w* Ch)) to reinforce Ch films. The authors determined that TS and EM values increased with the addition of CNC, for composites with 20% CNC revealing the optimal results. It was also noted that the introduction of nanoparticles increased the film crystallinity, which can help to explain the results.

Predominantly, chitosan matrixes reinforced with NC noted a growth in the tensile modulus and strength and a reverse decrease in the film elasticity. The nanofiller good dispersion in the chitosan matrix allied with the NC higher crystallinity, which confers strength and stiffness is essential to justify this reinforcement ability [51]. Moreover, in proper conditions, the reinforcement effect can be also explained by the connection of a well-dispersed NC within the polymeric array through hydrogen bonds. At low concentrations, NC demonstrates a considerable potential to develop the percolating stiff networks, due to the high availability of free hydroxyl groups [54,91]. The feasibility to constitute this mechanical improved network is directly connected with three fundamental features, the nanofiller dispersion, the NC aspect ratio, and the interaction strength between the nanofiller and matrix [92]. The study by Bras et al. (2011) [93] proved that NC with a higher aspect ratio improves the film tensile strength, resulting in a percolation threshold decrease. This allows the reduction of the necessary filler content to achieve an effective reinforcing effect [93]. A higher aspect ratio contributes to nanoparticles with a higher specific surface area, which could foment an enriched connection between the chitosan and NC, enabling a higher yield of stress transfer and restricting more efficiently the polymer chain’s motion and flow [81]. It should also be kept in mind that the source and the processes involved in the cellulose isolation and subsequent extraction of the nanocellulose affect its intrinsic characteristics, such as crystallinity, which consequently will equally influence its mechanical reinforcement capacity [51]. Additionally, the combination of other different factors present during the bionanocomposite elaboration can partly explain the differences found in the literature for the most appropriate compositions and condition range exposed from the Ch/NC bionanocomposites [53].

#### 2.2.3. Metal Oxides

Metal oxides (MO) are the most literature-referred nanoreinforcement in bionanocomposite structures for application in food packaging because, in addition to the mechanical reinforcement capacity, they also provide an extra antimicrobial efficiency to the bionanocomposite.

Among these metallic inorganic nanoreinforcements, titanium dioxide (TiO_2_) has captured significant attention because of its reasonable price, good stability, photocatalytic activity, UV resistance, antibacterial properties, and nontoxicity. Siripatrawan and Kaewklin (2018) [94] implemented four different concentrations of TiO_2_ (0.25%, 0.5%, 1%, and 2% (*w*/*w* Ch)) to reinforce chitosan films, and the mechanical properties were evaluated. The tensile strength efficacy increased with the nanoparticles introduction, with the most significant value being reached for 1% TiO_2_. The scanning electron microscope images showed that for higher concentrations, an aggregation seems to occur among the particles. The authors indicated that the aptness to agglomerate is presumably due to the decreasing range between the TiO_2_ nanoparticles through film-forming preparation, which contributes to the increasing the impact probability between them. Moreover, this study revealed that the EAB% decreases for Ch/TiO_2_ composites, but no contrast was recorded for different concentrations. Lan et al. (2021) [95] developed Ch/TiO_2_ films complemented with red apple pomace extract for multifunctional packaging material. The TS achieved by the composite with 0.5% (*w*/*w* Ch) TiO_2_ improved 21%, a value strikingly similar to the one in the previous study. When adding the apple extract this mechanical parameter improved, even more, having attained a 61% growth in relation to the pristine chitosan. In another work [96], a higher increase in tensile strength value, about 90%, was scored with fewer nanoparticle amounts, only 0.25% (*w*/*w* Ch). Curiously, against the observed trend lodged in the previous two studies, a greater increment in elongation was got, stretching more than 70% compared to the original chitosan film. To avoid the aggregation of the nanoparticles and consequently improve their dispersion in the biopolymeric matrix, Mallakpour and Madani (2015) [97] modified TiO_2_ with N-trimellitylimido-S-valine diacid to express biomaterial moieties through available amino acid groups. After achieving positive dispersion rates into the chitosan, the authors doubled the film TS with the introduction of 15% TiO_2_. 

Zinc oxide (ZnO) is another metal oxide broadly applied in the composite field due to its noticeable antimicrobial and photocatalytic properties. In parallel to other metal oxide nanoparticles, ZnO nanoparticles are appraised as safe for human beings and have been handled already as food additives and packaging materials [98]. In 2010, Li et al. [99] synthesized and characterized novel chitosan/ZnO composites membranes, in which the proper quantity of nanoparticles added was distributed in a broad range of values, between 1–10% (*w*/*w* Ch). The composite tensile strength was better for all ZnO amounts added comparatively with the original membrane; however, the lowest weight assigned was the one that produced the most extraordinary results. Predominantly, it was possible to understand that the mechanical properties of chitosan film only improved to ZnO with concentrations up to 3%. The authors explained that when reduced amounts of ZnO nanoparticles are released into the matrix, intermolecular hydrogen bonds faded and new Ch/ZnO hydrogen bonds were assembled, which created a more flexible molecular chain. Therefore, the film tensile strength and the elongation at break suffered a boost. Mujeeb Rahman et al. (2018) [100] also attributed the best reinforce percentage that should be added to chitosan films to 1% ZnO. With that amount, the authors reached more 225% in tensile strength than in the same film without nanoparticles. Other studies regarding ZnO reported the corresponding tendency verified for TiO_2_, and that lower amounts are more than enough to enhance the mechanical properties of the film, as can be seen by the results exposed in Table 7.

Other types of metal oxides, such as iron oxide (Fe_3_O_4_), silica oxide (SiO_2_), or magnesium oxide (MgO), have equally been used in interaction with chitosan, but more specifically in applications related to biomedical and effluent remediation, making it difficult to recognize results from its mechanical reinforcement. However, two quality studies can be found in the literature that directly applied MgO in a chitosan packaging system. Silva et al. (2017) [103] fabricated Ch/MgO thin films with improved physical properties for potential packaging application. Between the two MgO concentrations added (5% and 10% (*w*/*w* Ch)), the tensile strength and Young’s modulus were better for the lower amount, improving 86% and 38%, respectively. For both composites, the elasticity declined with the nanoparticles’ integration. Once again, the aggregation is the fundamental aspect to attribute better reinforcement to 5% with scanning electron microscopy (SEM) morphology showing that 10% MgO presented a relatively high number of micro-voids and pores. More recently, in 2020, Wang et al. [104] produced carboxymethyl chitosan (CMCS) and nano MgO for food packaging. In this case, the amount of MgO explored was much lower (0.5% and 1% (*w*/*w* Ch)) than in the previous study. Uncommonly, the authors perceived a decrease in tensile strength for both composites compared to chitosan film. In contrast, a greater increase was observed in elastic modulus and elongation at break. The authors concluded that the blend of CMCS and MgO might be used as a novel food packaging when ductility and elasticity are intended. Finally, Yadav et al. (2014) [105] added 0.5% (*w*/*w* Ch Fe_3_O_4_ to chitosan/graphene oxide (GO) composite. In comparison with the Ch/GO composite, the addition of iron oxide improved by 10% and 22%, in accordance with the TS and EM. 

As can be stated from Table 7, lower amounts of metal oxides should be enough to provide a better strength effect to chitosan films. By overtaking the threshold amount will occur nanoparticle aggregation and consequently that effect will vanish. Moreover, it should be noted that in these studies it is rare to find Young’s modulus results, being difficult to analyze the metal oxide nanoparticle effect in the composite stiffness. Regarding the elongation at break, a great disparity of results was demonstrated.

#### 2.2.4. Carbon Nanotubes

Carbon nanotubes (CNTs) are another nanoscale agent that have been labeled as ideal reinforcing filler for polymer matrixes to accomplish notable mechanical performance, because of their nanometer size, high aspect ratios, and more importantly, their remarkable strength and stiffness [106]. CNTs can be based in two different configurations, one-dimensional referred to as single-wall carbon nanotubes (SWCNTs) or two or more-dimensional assigned as multi-wall carbon nanotubes (MWCNTs) that understand nested single-wall carbon nanotubes, with this last type being widely used in the preparation of polymer nanocomposites [107]. Although the favorable characteristics to form functional bionanocomposites, the creation of a homogeneous Ch/CNT structure represents technical defiance due to the CNTs’ poor dispersion. The intrinsic van der Waals attractive forces cause carbon nanoparticles to be very susceptible to aggregation processes, which makes it impossible for an appropriate scattering, therefore affecting the CNT mechanical and physical properties. To surpass this obstacle, several physical and chemical methods have been proposed, such as CNT chemical functionalization, CNT dispersion in a polymer solution by an ultrasonic bath, or in situ polymerization [108]. Different examples of chitosan films reinforced with CNTs are stated in Table 8.

The modification of the CNT surface by functionalization of acidic groups is an effective method of preparing a uniform dispersion of CNT in a polymer matrix. The concentrated acids are used to form carboxylic acid groups on the CNT graphitic frame surface, which owns conjugated double bonds that could be oxidized. In 2005, Wang et al. (2005) [106] proposed a novel approach to prepare fully functionalized chitosan nanocomposites reinforced with carbon nanotubes by a simple solution-evaporation method. Their plan of action passed by formulating MWCNTs with multiple surface hydroxyl groups that will favor the blend with the chitosan amino bands through hydrogen bonding, improving the interfacial strength between the two components and consequently improving their dispersion into the polymeric matrix. The functionalized MWCNTs were prepared by refluxing in a mixture of concentrated sulfuric acid and nitric acid, producing carboxylic and hydroxyl groups. The achieved results incredibly demonstrated a significant increase of the matrix mechanical properties, in which it was observed that 0.8% (*w*/*w* Ch) of MWCNTs is enough to enhance the composite strength and stiffness above 90% when compared to those of neat Ch. On behalf of the robust interactions between Ch and MWCNT, higher forces were required to surmount the molecular bonding energy, and thereafter, elastic modulus and tensile strength increased. This methodology proved to be effective in forming a strong hydrogen bond with chitosan, but despite continuing to show good reinforcement rates, the change from 0.8% to 2% MWCNTs was not justified. The morphology images identified a nanoparticle aggregation, which can justify the stabilization of tensile strength for higher MWCNTs concentrations. Ke et al. (2007) [114] used a nucleophilic substitution reaction to create covalently functionalized carbon nanotubes. The covalent linkages shaped through the reaction between the Ch amino groups and the acyl chloride groups of the modified CNTs will promote more stable and effective blends. Nevertheless, the employed methodology included critical and risky steps to be performed. Two years later, Liu et al. [109] proposed an alternate and steadier approach to prepare chitosan/MWCNT nanocomposites using poly(styrene sulfonic acid) functionalized MWCNTs. The sulfonic acid groups generated supplied the necessary chemical reactivity and interfacial compatibility between MWCNT and Ch. The authors observed a significantly constant growth in TS and EM values with the increase of MWCNT added. For a nanoparticle concentration of 1.5% (*w*/*w* Ch), it was incredibly accomplished an increment of 162% and 384% for these parameters, revealing the vast capacity that modified MWCNTs may have to strengthen the Ch biofilm. In this academic study, there was an increase in the EAB% with the introduction of MWCNTs in the film, reaching double the elongation values for incorporations of nanoparticles above 1%. The small amount of water absorbed linked with the sulfonic acid groups added through hydrogen-bonds could contribute to the elongation growth. In addition to the nanoparticle distribution, a precise alignment of CNTs is crucial in the quality of the resultant nanocomposite, as stated by Ong et al. (2011) [110]. In their study, the chitosan matrix was reinforced with aligned poly(3-hydroxybutyrate) (PHB)-functionalized MWCNTs, in which the bulk alignment was accomplished using a simple filtration method. A chitosan composite with raw MWCNT was also produced to compare the effect of the functionalization. Comparing the two developed matrixes with pristine chitosan, it was possible to observe a significant mechanical difference between them. The raw-MWCNTs/Ch only lightly increases the TS and the EM values by 1.6% and 9.9%, respectively, while on the other hand, the presence of PHB–MWCNT considerably raises the TS of the chitosan matrix by 41.8% and increases the EM by 24.1%. In both membranes occurred a decrease in flexibility. Supporting the results previously exposed, these superior mechanical properties of the Ch/PHB–MWCNT nanocomposite membrane can be imputed to the suitable nanoparticle dispersion and their deep affinity with the chitosan matrix. Furthermore, the initial bulk alignment performer in the MWCNTs played an essential role in upgrading the nanocomposite mechanical properties. More recently, Wang et al. (2019) [113] developed high-performance chitosan membranes reinforced with polydopamine functionalized MWCNTs, followed by an ion crosslinking with sulfuric acid. The MWCNTs amounts added were superior to the other works, reaching 10%; however, it was for the 2% concentration that the greatest increase in TS and EAB% was observed.

As seen formerly, obtaining maximum compatibility between Ch and CNT is crucial in the nanocomposite preparation. Bearing that in mind, Iamsamai et al. (2010) [115] evaluated if the chitosan degree of deacetylation (%DD) sustained some kind of effect on the noncovalent surface modification of multiwall carbon nanotubes dispersion. For that purpose, 12.5 mg of MWCNTs were added to different solutions of chitosan comprising a set volume of 50 mL but distinct %DD (61, 71, 78, 84, 90, or 93). Zeta potential, centrifugation, and UV–Visible spectroscopy measurements suggested that the dispersion and stability of MWCNTs could be adequately improved when using chitosan with the lowest degree of deacetylation (61%DD) perhaps due to MWCNTs higher surface coverage. Although these data require mechanical tests to prove they are related, which was not provided by this scientific article, they can still be strong indicators. Bakhtiari et al. (2019) [112] combined four different chitosan percentages (1%, 1.5%, 2%, and 2.5% (*w*/*w* Ch)) as polymer matrix and two percentages (0.5% and 1% (*w*/*w* Ch)) of MWCNTs as reinforcement. As reported by the authors, the results of mechanical assays showed that the combination of 2.5% (*w*/*v*) Ch + 0.5% MWCNTs nanocomposite film registered the highest strength, which was increased by 176% (from 18.26 MPa to 50.46 MPa) and Young’s modulus film increased by 195% (from 406 MPa to 1197 MPa) when compared to the pristine Ch film. The higher concentration of Ch (2.5% *w*/*v*) in solution engenders more entangled Ch molecules and more connections to MWCNTs in parallel to a lower concentration.

Ensuing this chapter’s aim is also newsworthy to approach the synergy demonstrated between the carbon nanotubes and another nanofiller. Tang et al. (2009) [107] successfully prepared a chitosan ternary nanocomposite with two-dimensional MMTNa^+^ platelets and CNTs by solution-intercalation/mixing method in acid media. With dynamic mechanical measurement, the authors concluded that the chitosan/clay/carbon nanotube composite has better storage modulus than that for the corresponding binary chitosan/clay or chitosan/CNT nanocomposites with the identical filler content (3% (*w*/*w* Ch)). In two other studies, Bibi et al. [116] used a prompt and simple procedure to prepare functional chitosan nanocomposite films incorporating silver nanoparticles, gold nanoparticles, and CNTs with the support of ultrasound-assisted in situ synthesis. In both cases, a slight increase was observed in TS, toughness, and EAB% after CNTs inclusion in the composite, showing a good inference between different nanoparticles [116,117].

After exploring carbon nanotubes’ mechanical capacity, it is possible to deduce that in comparison to other analyzed nanoreinforcements, CNTs grant more tensile strength and elastic modulus with fewer particle concentrations. The good affinity these particles possess after some specific functionalization allows the composite to have high elongation rates. Furthermore, other factors such as good dispersion, alignment, and interaction with chitosan intrinsic parameters are essential to determine nanoparticle performance.

Other fillers in addition to nanoclays, cellulose nanofibers and nanocrystals, carbon nanotubes, and metal oxides are also being tested to prepare nanocomposites for food packaging applications. Yet, the information is still limited, and more research is needed in order to apprehend the significance of the current knowledge. Lignin, for example, is being considered by some authors as an option. Chitosan-based composite has been incorporated with lignin nano or macroparticles and their influence on the film’s structure for application as packaging films was investigated [118,119,120,121]. After cellulose, lignin is the second most abundant non-food component of biomass and is well-known for an overly complex structure. The richness of functional groups such as phenolic, carboxylic, and aliphatic hydroxyl groups could confer to the composite an increased antioxidant and antimicrobial activity. However, it is challenging to blend lignin with other polymers due to its brittle feature and pauper dispersion [118]. Rai et al. (2017) [118] isolated lignin from sugarcane bagasse, and then various fractions were added into chitosan solutions to acquire a final concentration of 100–400 mg/100 mL (*w*/*v*) in chitosan solution. The authors demonstrated a considerable decrease in tensile strength and elongation at break (%) with lignin incorporation, in which the lignin brittle characteristics could represent the response by the verified reduction in the film’s ductile properties. Jaganathan et al. (2018) [120] successfully isolated lignin from *Artocarpus heterophyllus* peels. In this particular study, an increase was observed in tensile strength, but elastic modulus and elongation at break (%) decreased with the introduction of lignin into the polymeric matrix. Regarding water vapor permeability, this study shows an increase of porosity and consequently higher values for this specific property for chitosan–lignin blends [120]. Therefore, more knowledge on the interaction of nanolignin with biopolymers, such as chitosan, can help to clarify the prospects of using this non-food component of biomass, contributing to the circular economy and bioeconomy.

## 3. Conclusions and Final Remarks

In this review, the analysis made on bionanocomposites characteristics allowed us to confirm that the incorporation of nanoparticles provides a significant reinforcement of the biopolymer’s barrier and mechanical properties. The type of nanocomposite, its aspect ratio, and the amount added to the chitosan film are crucial to determine the affinity established between both, and consequently, to obtain a good particle dispersion and percolation through the matrix. In the case of montmorillonite and nanocellulose, there seems to be a better reinforcement for concentrations between 2–5%, while for metal oxides and carbon nanotubes, it is observed that smaller amounts are sufficient to reach the same reinforcement values. This percentage allows achieving optimal nanofiller dispersion, while above these values, nanoparticles tend to aggregate, which will make films more brittle and more prone to gas diffusion. Another inference is that when the nanofillers are incorporated into the film, strong chemical bonds may be formed between the components; yet, in the case of carbon nanotubes, a functionalization is required to improve their effectiveness. Nonetheless, some other factors can explain discrepancies to the trends normally observed, e.g., the intrinsic chitosan features (degree of deacetylation and molecular weight), the addition of plasticizers (e.g., glycerol), natural extracts, essential oils, or crosslinkers. Furthermore, the technique employed to incorporate the nanoparticles, e.g., the application or not of ultrasounds bath homogenizer, can explain the differences registered between bionanocomposites with the same number of nanoparticles.

Present challenges toward the production and application of chitosan nanocomposites in the food packaging industry, on a large scale, are mainly related to their high costs when compared to the traditional petroleum-based plastics. But other challenges limit still its application. More knowledge is needed on the ecotoxicity and toxicity of the nanofillers being applied and on their potential migration to food. Additionally, ecofriendly, and more sustainable strategies for the production of nanofillers should be envisaged and other nanofillers could also be tested in order to identify promising options. This will provide more information on the factors that may hinder the full application of chitosan nanocomposites and other bionanocomposites in the food packaging industry and make it easier to achieve future developments in this area.

## Figures and Tables

**Table 1:** Prediction models for barrier properties of nanocomposites (adapted from [34]).

Model	Filler Type	Aspect Ratio (α)	Formula
Nielsen	Ribbon	α = w/t (length is infinite, w is the width, t is the thickness)	(P0/P)(1 − ϕ) = 1 + αϕ/2
Cussler (regular array)	Ribbon	α = w/t (length is infinite, w is the width, t is the thickness)	(P0/P)(1 − ϕ) = 1 + (αϕ)2/4
Cussler (random array)	Ribbon	α = w/t (length is infinite, w is the width, t is the thickness)	(P0/P)(1 − ϕ) = (1 + αϕ/3)2
Gusev and Lusti	Disk	α = d/t (circular shape, d is the diameter, t is the thickness)	(P0/P)(1 − ϕ) = exp[(αϕ/3.47)0.71]
Fredrickson and Bicerano	Disk	α = d/t (circular shape, d is the diameter, t is the thickness)	(P0/P)(1 − ϕ) = 4(1 + x + 0.1245x2)/(2 + x)2, where x = αϕ/2ln(α/2)
Bharadwaj	Disk	α = d/t (circular shape, d is the diameter, t is the thickness)	(P0/P)(1 − ϕ) = 1 + 0.667αϕ(S + 0.5), where S is the orientation factor from -0.5 to 1.

P—permeability of composites; P0—permeability of pure polymer; ϕ—volume fraction.

**Table 2:** Barrier properties of chitosan films reinforced with nanoclays.

Formulation	Incorporation Method	Water Vapor Permeability (Percentage Relatively to Control)	Oxygen Permeability (Percentage Relatively to Control)	Ref
2% (*w*/*v*) Ch (deacetylation degree of 75–85%); Glycerol 25% (*w*/*w* Ch)	Mechanical stirring and ultrasonic homogenizer	Ch + 1% MMTNa: Decreased 31% Ch + 3% MMTNa: Decreased 56% Ch + 5% MMTNa: Decreased 38%	Not Performed	[39]
1.5% (*w*/*v*) Ch (deacetylation degree of 75%); Glycerol 30% (*w*/*w* Ch)	Rotor–stator homogenizer (Ultra-Turrax) and ultrasonic homogenizer	Ch + 2.5% MMTNa: Increased 25%	Ch + 2.5% MMTNa: Decreased 47%	[25]
1% (*w*/*v*) Ch (deacetylation degree of about 75%); Glycerol 40% (*w*/*w* Ch)	Mechanical stirring and ultrasonic homogenizer	Ch + 5% HNT: Decreased 2% Ch + 10% HNT: Decreased 5% Ch + 15% HNT: Decreased 10% Ch + 20% HNT: Decreased 13% Ch + 25% HNT: Decreased 14% Ch + 30% HNT: Decreased 16%	Not Performed	[40]
2% (*w*/*v*) Ch (deacetylation degree greater than 75%); Glycerol 25% (*w*/*w* Ch)	Mechanical stirring and ultrasonic probes	Ch + 1% MMT: Decreased 28%	Ch + 1%MMT: Decreased 12%	[41]
		Ch + 3% MMT: Decreased 37%	Ch + 3%MMT: Decreased 55%	
		Ch + 5% MMT: Decreased 17%	Ch + 5%MMT: Decreased 37%	
		Ch + 1% MMTCuO-20: Decreased 26%	Ch + 1%MMTCuO-20: Decreased 31%	
		Ch + 3% MMTCuO-20: Decreased 39%	Ch + 3%MMTCuO-20: Decreased 60%	
		Ch + 5% MMTCuO-20: Decreased 35%	Ch + 5%MMTCuO-20: Decreased 49%	
		Ch + 1% MMTCuO-90: Decreased 72%	Ch + 1%MMTCuO-90: Decreased 47%	
		Ch + 3% MMTCuO-90: Decreased 77%	Ch + 3%MMTCuO-90: Decreased 79%	
		Ch + 5% MMTCuO-90: Decreased 76%	Ch + 5%MMTCuO-90: Decreased 55%	
1% (*w*/*w*) Ch (deacetylation degree greater than 85%); Glycerol 30% (*w*/*w* Ch) of chitosan	Nanoparticles were dispersed stirred at 400 rotational frequency and immersed in an ultrasound bath	Ch + 0.5% MMT: Increased 16%	Ch + 0.5% MMT: Increased 89%	[42]
		Ch + 1% MMT: Decreased 19%	Ch + 1% MMT: Increased 225%	
3% (*v*/*v*) Ch (deacetylation degree of about 95%); Polycaprolactone 12% (*w*/*w* Ch)	Mechanical stirring and ultrasonic homogenizer	Ch + 2% HNT: Decreased 15% Ch + 4% HNT: Decreased 23% Ch + 6% HNT: Decreased 25%	Not Performed	[43]
1.5% (*w*/*w*) Ch (deacetylation degree of about 90%)	Mechanical stirring and ultrasonic homogenizer	Ch + 0.5% MMTNa: Decreased 2.7%	Ch + 0.5% MMTNa: Decreased 32%	[37]
		Ch + 1% MMTNa: Decreased 7.4%	Ch + 1% MMTNa: Decreased 61%	
		Ch + 2% MMTNa: Decreased 4.0%	Ch + 2% MMTNa: Decreased 51%	
0.2% (*v*/*v*) Ch (deacetylation degree of about 75%); carboxymethyl cellulose 1% (*w*/*w* Ch)	Mechanical stirring	Ch + 5% HNT: Decreased 60% Ch + 7% HNT: Decreased 71% Ch + 10% HNT: Decreased 75%	Not Performed	[44]
2% (*w*/*w*) Ch (deacetylation degree of about 90%)	Mechanical stirring	Ch + 25% HNT: Decreased 6,1% Ch + 42% HNT: Decreased 15% Ch + 66% HNT: Decreased 29% Ch + 100% HNT: Decreased 20%	Not Performed	[45]

Ch—chitosan; MMTNa—sodium montmorillonite; HNT—halloysite nanotubes; MMTCuO—montmorillonite with copper (II) oxide.

**Table 3:** Barrier properties of chitosan films reinforced with cellulose nanoparticles.

Formulation	Incorporation Method	Water Vapor Permeability (Percentage Relatively to Control)	Oxygen Permeability (Percentage Relatively to Control)	Ref
2% (*w*/*v*) Ch (deacetylation degree not specified)	Mechanical stirring and ultrasonic homogenizer	Ch + 1% CNC: Decreased 34% Ch + 3% CNC: Decreased 16% Ch + 5% CNC: Decreased 38% Ch + 10% CNC: Decreased 25%	Not Performed	[53]
1% (*w*/*v*) Ch (deacetylation degree of 90%)	Mechanical stirring and ultrasonic homogenizer	Ch + 2% CNC: Decreased 24% Ch + 4% CNC: Decreased 29% Ch + 6% CNC: Decreased 34% Ch + 8% CNC: Decreased 37%	Not Performed	[54]
3% (*w*/*v*) Ch (degree of deacetylation 94%);	Mechanical stirring	Ch + 10% CNC: Decreased 32% Ch + 20% CNC: Decreased 28%	Not Performed	[55]
2% (*w*/*v*) Ch (deacetylation degree not specified)	Mechanical stirring	Ch + 10% CGTOCNF: Decreased 6%	Ch + 10% CGTOCNF: Increased 13%	[56]
		Ch + 17% CGTOCNF: Increased 2%	Ch + 17% CGTOCNF: Increased 16%	
		Ch + 25% CGTOCNF: Decreased 4%	Ch + 25% CGTOCNF: Increased 3%	
		Ch + 33% CGTOCNF: Decreased 7%	Ch + 33% CGTOCNF: Increased 62%	
1% (*w*/*v*) Ch (deacetylation degree 75–85%);	Nanoparticles were dispersed with the aid of mechanical stirring	Ch + 2% BCNC: Decreased 9% Ch + 4% BCNC: Decreased 20% Ch + 6% BCNC: Decreased 27%	Not Performed	[57]

Ch—chitosan; CNC—cellulose nanocrystals; CGTOCNF—curcumin-grafted; BCNC—bacterial nanocellulose.

**Table 4:** Barrier properties of chitosan films reinforced with metal oxide nanoparticles and carbon nanotubes.

Formulation	Incorporation Method	Water Vapor Permeability (Percentage Relatively to Control)	Oxygen Permeability (Percentage Relatively to Control)	Ref
1% (*w*/*v*) Ch; 2% (*w*/*v*) purified CNT in dimethylformamide; 6 different PLA/CNT /Ch concentrations (0, 1, 3, 5, 7, 9% Ch)	Nanotubes were dispersed with stirring and electrospinning	PLA/CNTs/Ch-1%: Decreased 30% PLA/CNTs/Ch-3%: Decreased 64% PLA/CNTs/Ch-5%: Decreased 71% PLA/CNTs/Ch-7%: Decreased 75% PLA/CNTs/Ch-9%: Decreased 54%	Not performed	[58]
1 g of Ch in the 1% acetic acid solution;	Metal oxides were sonicated to be dispersed	Ch + 0.1% ZnONP: Decreased 21% Ch + 0.3% ZnONP: Decreased 31% Ch + 0.5% ZnONP: Decreased 56%	Not performed	[59]
2% *(w*/*v*) Ch	Metal oxides were dispersed with stirring	Ch + 30ZnO: Decreased 66%	Ch + 30ZnO: Decreased 7.5%	[60]
		Ch + 50ZnO: Decreased 77%	Ch + 50ZnO: Decreased 10%	
		Ch + 70ZnO: Decreased 87%	Ch + 70ZnO: Decreased 41%	
0.5% (*w*/*v*) Ch; 1% cellulose acetate (CelAc) solution; 0.25% glycerol	Metal oxides were dispersed in formic acid and after were mixed with the polymer solution	Ch-CelAc-CeO_2_-0.1%: Increased 36% Ch-CelAc-CeO_2_-1%: Increased 157%	Not performed	[61]
Ch (0.2 g) was dispersed in 50.0 mL acidic water (0.5% *v*/*v* acetic acid	Metal oxides were dispersed in water and sonicated in an ultrasonic bath	CMC-Ch-OL-ZnONPs 0.5%: Increased 5.1% CMC-Ch-OL-ZnONPs 1%: Increased 20% CMC-Ch-OL-ZnONPs 2%: Increased 28%	Not performed	[14]
1.5% (*w/v*) Ch in a 1% (*v*/*v*) l-(β)-lactic acid; 0.5% glycerol (*w*/*v*); 3 different acetylene flux (4 sccm, 12 sccm, 20 sccm)	Carbon-based coatings were dispersed with radio-frequency reactive magnetron sputtering	Ch-Acetylene 4 sccm: Increased 34%	Ch-Acetylene 4 sccm: Increased 14%	[62]
		Ch-Acetylene 4 sccm: Increased 12%	Ch-Acetylene 12 sccm: Decreased 42%	
		Ch-Acetylene 4 sccm: Increased 11%	Ch-Acetylene 20 sccm: Decreased 81%	
1% (*w*/*v*) Ch; 20% glycerol (*w*/*v* Ch)	Metal oxides weredispersed with refluxed	ChG7ZnO: Decreased 81% (The other results are not showed)	Not performed	[63]
0.6% (*w*/*v*) Ch; 4 different ZnONP concentrations (0.165, 0.33, 0.66, 0.99g in 15 mL Water)	Metal oxides were dispersed with vigorous stirring	Ch/ZnO 0.165 g: Decreased 13% Ch/ZnO 0.33 g: Decreased 17% Ch/ZnO 0.66 g: Decreased 8.0% Ch/ZnO 0.99 g: Decreased 6.8%	Not performed	[64]
1% (*w*/*v*) Ch and 1% (*w*/*w* of Ch) of MgO NP	Metal oxides weredispersed with vigorous stirring	Decreased 53%	Not performed	[65]

Ch—chitosan; PLA—polylactic acid; CNTs—carbon nanotubes; ZnO—zinc Oxide; CelAc—cellulose acetate; CeO_2_—cerium (IV) oxide; CMC—carboxymethylcellulose; OL—oleic acid.

**Table 5:** Mechanical properties of chitosan films reinforced with nanoclays compared to pristine chitosan films.

Formulation	Incorporation Method	Tensile Strength (Relatively to Control)	Elongation at Break (Relatively to Control)	Elastic Modulus (Relatively to Control)	Ref
1.5% (*w*/*v*) Ch (deacetylation degree of 75–85%); Glycerol 40% (*w*/*w*) of chitosan	Mechanical stirring and ultrasonic homogenizer	1% MMTNa: Increased ~ 20–30%	1% MMTNa: Decreased ~ 5%	Not performed	[71]
		3% MMTNa: Increased ~ 50%	3% MMTNa: Decreased ~ 15%		
		5% MMTNa: Increased ~ 50%	5% MMTNa: Decreased ~ 20%		
		7% MMTNa:Increased ~ 20–30%	7% MMTNa:Decreased ~ 10–15%		
		9% MMTNa: Increased ~ 20–30%	9% MMTNa: Decreased ~ 10–15%		
		11% MMTNa: Increased ~ 20–30%	11% MMTNa: Decreased ~ 10–15%		
2% (*w*/*v*) Ch (deacetylation degree of 88.8%); Glycerol 40% (*w*/*w*) of chitosan	Mechanical stirring and rotor–stator homogenizer (Ultra-Turrax)	1% MMTNa: Increased ~ 5%	1% MMT: Decreased ~ 5%	Not performed	[72]
		3% MMTNa: Increased ~ 20%	3% MMT: Decreased ~ 19%		
		5% MMTNa: Increased ~ 15%	5% MMT: Decreased ~ 14%		
		1% MMTNa + 0.5% REO: Increased ~ 18%	1% MMTNa + 0.5%REO: Increased ~ 18%		
		3% MMTNa + 0.5% REO: Increased ~ 21%	3% MMTNa + 0.5% REO: Increased ~ 21%		
		5% MMTNa + 0.5% REO: Increased ~ 37%	5% MMTNa + 0.5% REO: Increased ~ 37%		
1% and 2% (*w*/*v*) Ch (deacetylation degree of 75–85%); Glycerol 30% (*w*/*w*) of chitosan	Reflux-solution method	1% Ch + 3% MMTNa: Increased ~ 3%	1% Ch + 3% MMTNa: Decreased ~ 64%	1% Ch + 3% MMTNa: Increased ~ 21%	[74]
		1% Ch + 5% MMTNa: Increased ~ 17%	1% Ch + 5% MMTNa: Decreased ~ 38%	1% Ch + 5% MMTNa: Increased ~ 35%	
		1% Ch + 10%MMTNa: Increased ~ 19%	1% Ch + 10% MMTNa: Decreased ~ 60%	1% Ch + 10%MMTNa: Increased ~ 27%	
		2% Ch + 3% MMTNa:Increased ~ 69%	2% Ch + 3% MMTNa: Decreased ~ 61%	2% Ch + 3% MMTNa:Increased ~ 100%	
		2% Ch + 5% MMTNa: Increased ~ 22%	2% Ch + 5% MMTNa: Decreased ~ 68%	2% Ch + 5% MMTNa: Increased ~ 43%	
		2% Ch + 10% MMTNa: Decreased ~ 1%	2% Ch + 10% MMTNa: Decreased ~ 75%	2% Ch + 10% MMTNa: Increased ~ 80%	
1% (*w*/*v*) Ch (deacetylation degree greater than 85%); Glycerol 30% (*w*/*w*) of chitosan	Mechanical stirring and ultrasonic homogenizer	0.5% MMTNa: Decreased ~ 75%	0.5% MMTNa: Increased ~ 20%	0.5%MMTNa: Decreased ~ 98%	[42]
		1% MMTNa: Decreased ~ 57%	1%MMTNa: Increased ~ 23%	1%MMTNa: Decreased ~ 97%	
1.5% (*w*/*v*) Ch (deacetylation degree of 75%); Glycerol 30% (*w*/*w*) of chitosan	Rotor–stator homogenizer (Ultra-Turrax) and ultrasonic bath	2.5% MMTNa: Increased ~ 82%	2.5% MMTNa: Decreased ~ 52%	2.5% MMTNa: Increased ~ 137%	[75]
		2.5% MMTCa: Increased ~ 70%	2.5% MMTCa: Decreased ~ 65%	2.5% MMTCa: Increased ~ 184%	
		2.5% MMT20: Increased ~ 70%	2.5% MMT20: Decreased ~ 23%	2.5% MMT20: Increased ~ 110%	
1.5% (*w*/*v*) Ch (deacetylation degree of 75%); Glycerol 30% (*w*/*w*) of chitosan	Rotor–stator homogenizer (Ultra-Turrax) and ultrasonic homogenizer	2.5% MMTNa + 0.5% GEO: Decreased ~ 10%	2.5% MMTNa + 0,5% GEO: Increased ~ 25%	2.5% MMTNa+0.5%GEO: Decreased ~ 37%	[25]
		2.5% MMTNa + 1% GEO: Decreased ~ 26%	2.5% MMTNa + 1% GEO: Increased~ 85%	2.5% MMTNa + 1% GEO: Decreased ~ 57%	
		2.5% MMTNa+2%GEO: Decreased ~ 35%	2.5% MMTNa + 2% GEO: Increased ~ 100%	2.5% MMTNa + 2% GEO: Decreased ~ 77%	
2% (*w*/*v*) Ch (deacetylation degree of 75–85%)	Reflux-solution method	5% MMTNa: Increased ~ 16%	5% MMTNa: Decreased ~ 48%	5% MMTNa: Increased ~ 20%	[76]
		5% OrgMMT: Increased ~ 14%	5% OrgMMT: Decreased ~ 15%	5% OrgMMT: Increased ~ 18%	
		5% MMTNa + TO: Increased ~ 9%	5% MMTNa + TO: Decreased ~ 19%	5% MMTNa+TO: Increased ~ 2%	
		5% OrgMMT + TO: Increased ~ 11%	5% OrgMMT + TO: Decreased ~ 54%	5% OrgMMT+TO: Increased ~ 6%	

Ch—chitosan; MMTNa—sodium montmorillonite; REO—rosemary essential oil; MMTCa—calcium montmorillonite; GEO—ginger essential oil; OrgMMT—organophilic montmorillonite; TO—thyme oil.

**Table 6:** Mechanical properties of chitosan films reinforced with nanocellulose compared to pristine chitosan films.

Formulation	Incorporation Method	Tensile Strength (Relatively to Control)	Elongation at Break (Relatively to Control)	Elastic Modulus (Relatively to Control)	Ref
1% (*w*/*v*) Ch (deacetylation degree of 88%)	Rotor–stator homogenizer (Ultra-Turrax)	1% CNC: Increased ~ 9%	1% CNC: Decreased ~ 25%	1% CNC: Increased ~ 43%	[91]
		3% CNC: Increased ~ 17%	3% CNC: Decreased ~ 40%	3% CNC: Increased ~ 60%	
		5% CNC: Increased ~ 25%	5% CNC: Decreased ~ 55%	5% CNC: Increased ~ 87%	
		10% CNC: Increased ~ 24%	10% CNC: Decreased ~ 55%	10% CNC: Increased ~ 80%	
2% (*w*/*v*) Ch (deacetylation degree not specified)	Mechanical stirring and ultrasonic homogenizer	Ch + 1% CNC: Increased ~ 1%	Ch + 1% CNC: Decreased ~ 33%	Ch + 1% CNC: Increased ~ 13%	[53]
		Ch + 3% CNC: Decreased ~ 3%	Ch + 3% CNC: Decreased ~ 14%	Ch + 3% CNC: Increased ~ 47%	
		Ch + 5% CNC: Increased ~ 18%	Ch + 5% CNC: Increased ~ 24%	Ch + 5% CNC: Increased ~ 32%	
		Ch + 10% CNC: Increased ~ 8%	Ch + 10% CNC: Increased ~ 28%	Ch + 10% CNC: Increased ~ 2%	
1% (*w*/*v*) Ch (deacetylation degree of 98%)	Mechanical stirring and Silent Crusher-Heidolph homogenizer	Ch + 5% CNC: Increased ~ 9%	5% CNC: Increased ~ 22%	Ch + 1% CNC: Increased ~ 7%	[84]
		Ch + 10% CNC: Increased ~ 11%	10% CNC: Decreased ~ 2%	Ch + 3% CNC: Increased ~ 22%	
		Ch + 20% CNC: Increased ~ 24%	20% CNC: Decreased ~ 5%	Ch + 5% CNC: Increased ~ 140%	
		Ch + 30% CNC: Increased ~ 17%	30% CNC: Increased ~ 1%	Ch + 10% CNC: Increased ~ 86%	
4% (*w*/*v*) Ch (deacetylation degree of 90%)	Mechanical stirring and ultrasonic homogenizer	1% CNC: Increased ~ 5%	1% CNC: Decreased ~ 15%	Not Performed	[86]
		3% CNC: Increased ~ 30%	3% CNC: Decreased ~ 20%		
		5% CNC: Increased ~ 71%	5% CNC: Decreased ~ 20%		
		8% CNC: Increased ~ 52%	8% CNC: Decreased ~ 35%		
		10% CNC: Increased ~ 46%	10% CNC: Decreased ~ 40%		
		15% CNC: Increased ~ 23%	15% CNC: Decreased ~ 50%		
		20% CNC: Increased ~ 13%	20% CNC: Decreased ~ 65%		
1% (*w*/*v*) Ch (deacetylation degree of 90%)	Mechanical stirring and ultrasonic homogenizer	Ch + 2% CNC: Increased ~ 5%	Ch + 2% CNC: Decreased ~ 23%	Ch + 2%CNC: Increased ~ 39%	[54]
		Ch + 4% CNC: Increased ~ 39%	Ch + 4% CNC: Decreased ~ 55%	Ch + 4%CNC: Increased ~ 79%	
		Ch + 6% CNC: Increased ~ 35%	Ch + 6% CNC: Decreased ~ 58%	Ch + 6%CNC: Increased ~ 72%	
		Ch + 8% CNC: Increased ~ 32%	Ch + 8% CNC: Decreased ~ 59%	Ch + 8% CNC: Increased ~ 69%	
Not specified % (*w*/*v*) Ch (deacetylation degree of 90%)	Mechanical stirring and ultrasonic homogenizer	Ch + 1% CNF: Increased ~ 10%	Ch + 1% CNF: Decreased ~ 8%	Ch + 1% CNF: Increased ~ 7%	[88]
		Ch + 3% CNF: Increased ~ 22%	Ch + 3% CNF: Decreased ~ 18%	Ch + 3% CNF: Increased ~ 14%	
		Ch + 5% CNF: Increased ~ 31%	Ch + 5% CNF: Decreased ~ 28%	Ch + 5% CNF: Increased ~ 18%	
		Ch + 7% CNF: Increased ~ 25%	Ch + 7% CNF: Decreased ~ 22%	Ch + 7% CNF: Increased ~ 14%	
1% (*w*/*v*) Ch (deacetylation degree of 75–80%)	Mechanical stirring and ultrasonic homogenizer	Ch + 1%CNF: Increased ~ 31%	Ch + 1% CNF: Decreased ~ 10%	Ch + 1% CNF: Increased ~ 20%	[89]
		Ch + 3%CNF: Increased ~ 87%	Ch + 3% CNF: Decreased ~ 15%	Ch + 3% CNF: Increased ~ 43%	
		Ch + 5% CNF: Increased ~ 126%	Ch + 5% CNF: Decreased ~ 19%	Ch + 5% CNF: Increased ~ 89%	
		Ch + 7% CNF: Increased ~ 92%	Ch + 7% CNF: Decreased ~ 37%	Ch + 7% CNF: Increased ~ 51%	
1% (*w*/*v*) Ch (deacetylation degree of 75%)	Mechanical stirring and ultrasonic homogenizer	Ch + 2% BCNC: Increased ~ 28%	Ch + 2% BCNC: Decreased ~ 12%	Ch + 2% BCNC: Increased ~ 95%	[57]
		Ch + 4% BCNC: Increased ~ 96%	Ch + 4% BCNC: Decreased ~ 30%	Ch + 4% BCNC: Increased ~ 206%	
		Ch + 6% BCNC: Increased ~ 65%	Ch + 6% BCNC: Decreased ~ 26%	Ch + 6% BCNC: Increased ~ 119%	

Ch—chitosan; CNC—cellulose nanocrystals; CNF—cellulose nanofibers; BCNC—bacterial nanocellulose.

**Table 7:** Mechanical properties of chitosan films reinforced with metal oxides compared to pristine chitosan films.

Formulation	Incorporation Method	Tensile Strength (Relatively to Control)	Elongation at Break (Relatively to Control)	Elastic Modulus (Relatively to Control)	Ref
2% (*w*/*v*) Ch (deacetylation degree of 75–85%)	Mechanical stirring and ultrasonic homogenizer	5% TiO_2_: Increased ~ 40%	5% TiO_2_: Increased ~ 35%	Not Performed	[97]
		10% TiO_2_: Increased ~ 63%	10% TiO_2_: Decreased ~ 6%		
		15% TiO_2_: Increased ~ 100%	15% TiO_2_: Decreased ~ 10%		
2.5% (*w*/*v*) Ch (deacetylation degree of 90%)	Mechanical stirring	0.25% TiO_2_: Increased ~ 90%	0.25%TiO_2_: Increased ~ 70%	Not Performed	[96]
2% (*w*/*v*) Ch (deacetylation degree of 85%)	Controlled-temperature water bath shaker and ultrasonic homogenizer	0.25% TiO_2_: Increased ~ 10%	0.25% TiO_2_: Decreased ~ 5–15%	Not Performed	[94]
		0.5% TiO_2_: Increased ~ 20%	0.5% TiO_2_: Decreased ~ 5–15%		
		1% TiO_2_: Increased ~ 50%	1% TiO_2_: Decreased ~ 5–15%		
		2% TiO_2_: Increased ~ 25%	2% TiO_2_: Decreased ~ 5–15%		
2% (*w*/*v*) Ch (deacetylation degree of 90%)	Mechanical stirring	0.5% TiO_2_: Increased ~ 21%	0.5% TiO_2_: Decreased ~ 9%	Not Performed	[101]
		0.5% TiO_2_ + BPPE: Increased ~ 61%	0.5% TiO_2_+0.5 BPPE%: Increased ~ 6%		
2.5% (*w*/*v*) Ch (deacetylation degree of 85%)	Mechanical stirring	10% TiO_2_: Increased ~ 40%	10% TiO_2_: Increased ~ 15%	Not Performed	[95]
		10% TiO_2_+APE: Increased ~ 70%	10% TiO_2_+5%APE: Increased ~ 22%		
1% (*w/v*) Ch (deacetylation degree of 85%)	Mechanical stirring and ultrasonic homogenizer	1% ZnO: Increased ~ 83%	1% ZnO: Increased ~ 138%	Not Performed	[99]
		2% ZnO: Increased ~ 78%	2% ZnO: Increased ~ 62%		
		3% ZnO: Increased ~ 70%	3% ZnO: Increased ~ 36%		
		4% ZnO: Increased ~ 68%	4% ZnO: Decreased ~ 12%		
		5% ZnO: Increased ~ 54%	5% ZnO: Decreased ~ 33%		
		6% ZnO: Increased ~ 24%	6% ZnO: Decreased ~ 47%		
		7% ZnO: Increased ~ 19%	7% ZnO: Decreased ~ 59%		
		10% ZnO: Increased ~ 16%	10% ZnO: Decreased ~ 61%		
Not specified % (*w*/*v*) Ch (deacetylation degree unknown)	Mechanical stirring and ultrasonic homogenizer	0.1% ZnO: Increased ~ 15%	0.1% ZnO: Increased ~ 30%	Not Performed	[59]
		0.3% ZnO: Increased ~ 50%	0.3% ZnO: Increased ~ 75%		
		0.5% ZnO: Increased ~ 70%	0.5% ZnO: Increased ~ 90%		
		0.5% ZnO + NEO: Increased ~ 100%	0.5% ZnO + NEO: Increased ~ 130%		
2% (*w*/*v*) Ch (deacetylation degree of >75%)	Mechanical stirring	1% ZnO: Increased ~ 32%	1% ZnO: Decreased ~ 18%	1% ZnO: Increased ~ 47%	[102]
		2% ZnO: Increased ~ 67%	2% ZnO: Decreased ~ 57%	2% ZnO: Increased ~ 81%	
2% (*w*/*v*) Ch (deacetylation degree of 85%)	Ultrasonic homogenizer	0.5% ZnO: Increased ~ 103%	0.5% ZnO: Decreased ~ 26%	Not Performed	[100]
		1% ZnO: Increased ~ 225%	1% ZnO: Increased ~ 7%		
		1.5% ZnO: Increased ~ 182%	1.5% ZnO: Decreased ~ 52%		
		2% ZnO: Increased ~ 122%	2% ZnO: Decreased ~ 56%		
Not specified % (*w*/*v*) Ch (deacetylation degree of >75%)	Reflux-solution method	3% ZnO: Decreased ~ 25%	3% ZnO: Decreased ~ 3%	3% ZnO: Decreased ~ 42%	[63]
		5% ZnO: Decreased ~ 20%	5% ZnO: Increased ~ 35%	5% ZnO: Decreased ~ 40%	
		7% ZnO: Decreased ~ 15%	7% ZnO: Increased ~ 65%	7% ZnO: Decreased ~ 43%	
1% (*w*/*v*) Ch (deacetylation degree of 75%)	Mechanical stirring and ultrasonic homogenizer	5% MgO: Increased ~ 86%	5% MgO: Decreased ~ 13%	5% MgO: Increased ~ 38%	[103]
		10% MgO: Increased ~ 26%	10% MgO: Decreased ~ 25%	10% MgO: Increased ~ 27%	
1% (*w*/*v*) CMCh (deacetylation degree of 90%)	Mechanical stirring and ultrasonic homogenizer	0.5% MgO: Decreases ~ 30–35%	0.5% MgO: Increased ~ 142%	0.5% MgO: Increased ~ 24%	[104]
		1% MgO: Decreased ~ 10–15%	1% MgO: Increased ~ 171%	1% MgO: Increased ~ 88%	

Ch—chitosan; TiO_2_—titanium dioxide; BPPE—black plum peel extract; APE—red apple pomace; ZnO—zinc Oxide; NEO—neem oil; MgO—magnesium oxide.

**Table 8:** Mechanical properties of chitosan films reinforced with carbon nanotubes compared to pristine chitosan films.

Formulation	Incorporation Method	Tensile Strength (Relatively to Control)	Elongation at Break (Relatively to Control)	Elastic Modulus (Relatively to Control)	Ref
1% (*w*/*v*) Ch (deacetylation degree of 83%)	Rotor–stator homogenizer (Ultra-Turrax) and ultrasonic homogenizer	0.2% MWCNT: Increased ~ 23%	0.2% MWCNT: Decreased ~ 27%	0.2% MWCNT: Increased ~ 49%	[106]
		0.4% MWCNT: Increased ~ 78%	0.4% MWCNT: Decreased ~ 58%	0.4% MWCNT: Increased ~ 94%	
		0.8% MWCNT: Increased ~ 93%	0.8% MWCNT: Decreased ~ 61%	0.8% MWCNT: Increased ~ 99%	
		2% MWCNT: Increased ~ 99%	2% MWCNT: Decreased ~ 73%	2% MWCNT: Increased ~ 97%	
3% (*w*/*v*) Ch (deacetylation degree of 85%)	Mechanical stirring	0.1% MWCNT: Increased ~ 25%	0.1% MWCNT: Increased ~ 45%	0.1% MWCNT: Increased ~ 136%	[109]
		0.5% MWCNT: Increased ~ 59%	0.5% MWCNT: Increased ~ 95%	0.5% MWCNT: Increased ~ 288%	
		1%MWCNT: Increased ~ 119%	1% MWCNT: Increased ~ 150%	1% MWCNT: Increased ~ 322%	
		1.5%MWCNT: Increased ~ 162%	1.5% MWCNT: Increased ~ 200%	1.5%MWCNT: Increased ~ 384%	
Not specified % (*w*/*v*) Ch (deacetylation degree of 75–85%)	Coating	0.1% Raw-MWCNT: Increased ~ 2%	0.1%Raw-MWCNT: Decreased ~ 6%	0.1%Raw-MWCNT: Increased ~ 10%	[110]
		0.1% PHB–MWCNT: Increased ~ 42%	0.1% PHB–MWCNT: Increased ~ 28%	0.1% PHB–MWCNT: Increased ~ 24%	
2% (*w*/*v*) Ch (deacetylation degree of 75–85%)	Mechanical stirring and ultrasonic homogenizer	0.1%MWCNT: Increased ~ 10%	0.1% MWCNT: Decreased ~ 15%	0.1% CNT: Increased ~ 10%	[111]
		0.5% MWCNT: Increased ~ 20%	0.5% MWCNT: Decreased ~ 30%	0.5% CNT: Increased ~ 40%	
		0.5% MWCNT: Increased ~ 20%	1% MWCNT: Decreased ~ 33%	1% CNT: Increased ~ 47%	
1, 1.5, 2, and 2.5% (*w*/*v*) Ch (deacetylation degree of 75–85%)	Mechanical stirring and ultrasonic homogenizer	1% Ch + 0.5% MWCNT: Increased ~ 44%	Not Performed	1% Ch + 0.5% MWCNT: Increased ~ 19%	[112]
		1% Ch + 1% MWCNT: Increased ~ 31%		1% Ch + 1% MWCNT: Increased ~ 5%	
		1.5% Ch + 0.5% MWCNT: Increased ~ 66%		1.5% Ch + 0.5% MWCNT: Increased ~ 72%	
		1.5%Ch+1%MWCNT: Increased ~ 41%		1.5% Ch + 1% MWCNT: Increased ~ 55%	
		2% Ch + 0.5% MWCNT: Increased ~ 113%		2% Ch + 0.5% MWCNT: Increased ~ 143%	
		2% Ch + 1% MWCNT: Increased ~ 84%		2% Ch + 1% MWCNT: Increased ~ 70%	
		2.5% Ch + 0.5% MWCNT: Increased ~ 176%		2.5% Ch + 0.5% MWCNT: Increased ~ 195%	
		2.5% Ch + 1% MWCNT: Increased ~ 139%		2.5% Ch + 1% MWCNT: Increased ~ 77%	
2% (*w*/*v*) Ch (deacetylation degree of 92.5%)	Mechanical stirring and ultrasonic homogenizer	1% MWCNT: Increased ~ 6%	1% MWCNT: Increased ~ 57%	Not Performed	[113]
		2% MWCNT: Increased ~ 47%	2% MWCNT: Increased ~ 70%		
		5% MWCNT: Increased ~ 17%	5% MWCNT: Increased ~ 34%		
		10% MWCNT: Increased ~ 23%	10% MWCNT: Increased ~ 53%		

Ch—chitosan; MWCNT—multi-wall carbon nanotubes; PHB—poly(3-hydroxybutyrate).
